# Avoiding costly mistakes in groups: The evolution of error management in collective decision making

**DOI:** 10.1371/journal.pcbi.1010442

**Published:** 2022-08-19

**Authors:** Alan N. Tump, Max Wolf, Pawel Romanczuk, Ralf H. J. M. Kurvers

**Affiliations:** 1 Center for Adaptive Rationality, Max Planck Institute for Human Development, Berlin, Germany; 2 Science of Intelligence, Technische Universität Berlin, Berlin, Germany; 3 Leibniz Institute of Freshwater Ecology and Inland Fisheries, Berlin, Germany; 4 Institute for Theoretical Biology, Department of Biology, Humboldt Universität zu Berlin, Berlin, Germany; 5 Bernstein Center for Computational Neuroscience Berlin, Berlin, Germany; Peking University, CHINA

## Abstract

Individuals continuously have to balance the error costs of alternative decisions. A wealth of research has studied how single individuals navigate this, showing that individuals develop response biases to avoid the more costly error. We, however, know little about the dynamics in groups facing asymmetrical error costs and when social influence amplifies either safe or risky behavior. Here, we investigate this by modeling the decision process and information flow with a drift–diffusion model extended to the social domain. In the model individuals first gather independent personal information; they then enter a social phase in which they can either decide early based on personal information, or wait for additional social information. We combined the model with an evolutionary algorithm to derive adaptive behavior. We find that under asymmetric costs, individuals in large cooperative groups do not develop response biases because such biases amplify at the collective level, triggering false information cascades. Selfish individuals, however, undermine the group’s performance for their own benefit by developing higher response biases and waiting for more information. Our results have implications for our understanding of the social dynamics in groups facing asymmetrical errors costs, such as animal groups evading predation or police officers holding a suspect at gunpoint.

## Author summary

Decision makers must continuously balance the error costs of alternative decisions, especially in critical situations where the choices are associated with highly asymmetric error costs—such as pedestrian groups crossing the street, or animal groups evading predation. Acting independently, individuals can develop response biases to avoid more costly errors (e.g., when in doubt, escape from a potential predator). In groups, early decisions can spread via social influence and promote safe or risky behavior. Yet, little is known about how individuals in groups avoid the more costly error. We investigate this by modeling the decision process of individuals in groups using an evidence accumulation model. We derive the optimal strategies under different error costs, group sizes and pay off functions. We find that cooperative individuals in large groups do not evolve response biases because such biases rapidly amplify in groups. However, selfish individuals evolve high response biases and wait longer for the information of others, thereby undermining the group’s performance for own benefits. Our results shed light on the decision problems individuals in groups face in the presence of asymmetric error costs and how they could resolve them.

## Introduction

Individuals must continuously balance the error costs of alternative decisions. Is it, for example, better to escape or stay given a noisy cue indicating the potential presence of a predator; better to eat this new fruit or continue searching; or better to cross the red light or wait till it turns green? A fundamental characteristic of most decision-making environments is that the costs of different errors are not symmetrical [1–5]. For example, mistakenly identifying a stick as a snake is largely harmless, whereas believing a snake is a stick can be a lethal mistake. It is therefore crucial to incorporate such error cost asymmetries into accounts of adaptive decision making. A large body of literature (e.g., signal detection theory and error management theory) has investigated how individuals adjust their decision-making strategies to differences in error cost (or base rate) asymmetries, showing that individuals facing asymmetric costs develop a response bias—an increase in the probability of choosing a particular option—to avoid the more costly error [5–9]. However, the question of how groups deal with asymmetric error costs has received far less attention, even though individuals in collective systems often face highly asymmetric costs—for instance, animal groups under predation risk have to balance the risk of needlessly escaping versus getting predated [10], pedestrian groups crossing a busy street have to balance the risk of long waiting times versus a potential accident [11], and police officers holding a suspect at gunpoint have to trade-off the risk of shooting an unarmed suspect versus getting shot [12]. In collective systems, individual choices can spread via social influence through the group and steer others towards or away from making the more costly error; the consequences can be devastating for both individuals and the group (or society) as a whole. Understanding the dynamics of these cascades, in particular when and why they go wrong (e.g., deadly crowd panics or police officers shooting unarmed suspects), is thus important for a wide range of social systems.

Previous works have investigated collective decision making under asymmetric error costs, but used highly idealized decision-making processes. Wolf et al. studied how to optimally pool information in a collective decision-making scenario where individuals simultaneously indicated their personal decision (i.e., independent voting) in a binary classification task [13]. In binary classification, individuals categorise the world into one of two possible states: signal (e.g., a predator, disease, or other threat) or no signal. In doing so, they trade off between two possible types of error: misses (incorrectly deciding a signal is absent) and false alarms (incorrectly deciding a signal is present; [6, 9, 14, 15]). Wolf et al. [13] showed that in the presence of a response bias, optimal decisions arise when individuals do not simply follow the majority but instead set a quorum threshold between the true and false positive rate of their group members. Building on this, [4] showed that such quorum thresholds are extremely powerful for optimizing decision making across a broad range of environmental conditions (see also [16]).

The aforementioned studies assumed that all group members announce their personal decisions independently, and that individuals have access to the group’s average opinion as social information. Both assumptions are, however, unrealistic for decision-making processes in almost all biological systems (e.g., [17]). Rather, individuals often observe the choices (or actions) of others to inform their own decisions. Thus, they decide sequentially whereby early decisions can influence later-deciding individuals, and, in extreme cases, can trigger information cascades in which all other individuals imitate these early decisions [18–21]. Sequential decision making allows individuals to coordinate their (timing of) actions, with previous research showing that more knowledgeable (or confident) individuals generally make faster decisions and promote the spread of accurate information to less knowledgeable (or confident) followers [22–26]. Both accurate leaders and less accurate followers can benefit from such self-organisation as leaders save time and avoid potentially misleading social information, while followers may benefit from accurate social information.

**Fig 1:**
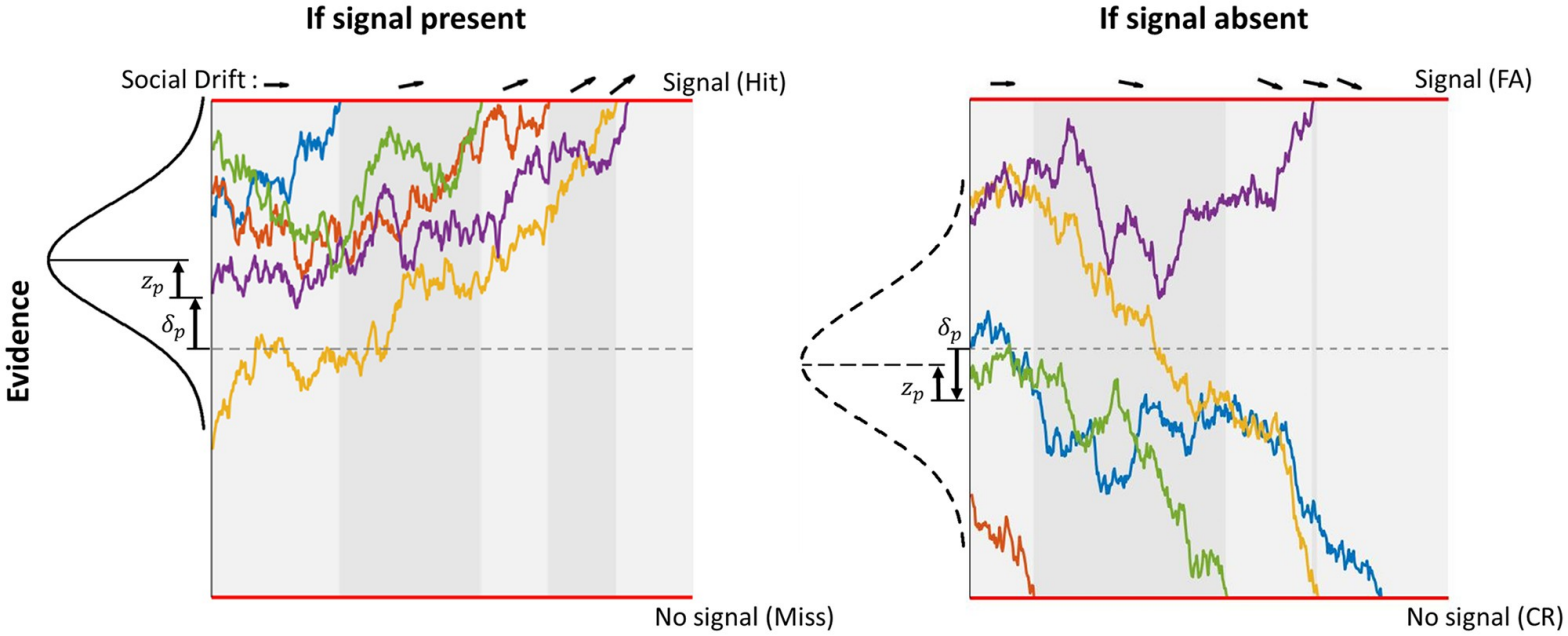
Illustration of the social drift–diffusion model. Each individual, represented by a jagged line, must decide whether a signal is present (left panel) or absent (right panel). If the signal is present, the individual can decide correctly (hit) or wrongly (miss). If the signal is absent, the individual can decide correctly (correct rejection; CR) or wrongly (false alarm; FA). An individual’s start point depends on the information it gathered prior to the social phase (*δ*_*p*_) and its start point bias (*z*_*p*_). Here the start point bias is towards the decision boundary (i.e., the red horizontal line) of the signal, implying that an individual is more likely to make a correct (wrong) decision when the signal is present (absent). At the start, no individual reached either decision boundary, implying that social information was absent. As individuals diffuse they hit a decision boundary and make a decision. Undecided individuals, in turn, start drifting towards the choice of the individual(s) that already decided, reflecting the process of social information use.

A general framework for modeling such realistic group dynamics are evidence accumulation models. These are an extension of signal detection theory models but can additionally account for the strategic decision timing of individuals [27]. These models propose that individuals accumulate evidence until there is sufficient evidence for one of the available options, triggering a decision. Evidence accumulation models, and particularly its most prominent member, the drift-diffusion model (DDM), have been very successful in shedding light on the cognitive underpinnings of a wide range of individual-level decision problems, such as predator avoidance [28], speed-accuracy trade-off [29], memory retrieval [30] and the influence of attention [31]. Recently, the DDM has also been applied to model information flow in groups [25, 32–35]. As individuals’ decision timing plays a key role in the unfolding collective dynamics, DDMs are ideally placed to model such dynamic collective processes because they explicitly model choice and timing. Thereby, they can account for the integration of personal and social information dynamically over time in a single framework. Analysing collective patterns by accounting for cognitive components of the decision–making process is a promising approach to understand realistic dynamic social interactions [36, 37]. Here we utilize the DDM’s ability to embody a realistic decision–making scenario of sequential decision making to study adaptive strategies under asymmetric costs by implementing a social version of the DDM (Fig 1).

In our implementation of the social DDM, each individual first accumulates its own personal information about the state of the world. During the social phase, individuals can gather additional social information (i.e., they can incorporate the choices of others). When an individual has gathered sufficient evidence (i.e., the decision boundary is exceeded), the decision is made. Because individuals both emit and receive social information, the system is highly dynamic and the final outcome is likely to be influenced by early responders with strong evidence. To examine how individuals should strategically adjust decision–making traits to the environment, we retrieved the adaptive behavioral parameters using evolutionary algorithms. Evolutionary algorithms allow to find fitness-maximizing behaviour by exposing the underlying behavioural traits to selection pressure, letting the traits evolve in concert [38]. We systematically varied group size and the asymmetry in error costs in order to study their combined effect on the evolution of the three key decision-making traits: start point bias, social information use, and the amount of evidence needed to decide.

We started with groups of individuals whose interests were completely aligned, with individuals equally sharing a group payoff (“cooperative groups”). We then investigated whether the collective interest was at odds with individuals’ self-interest (i.e., a social dilemma; [39]), by examining how introducing individual-level competition (i.e., a payoff solely based on own performance) shaped evolved behaviors and corresponding payoffs across group sizes and error cost asymmetries.

Our results show that in small cooperative groups facing high asymmetric costs, individuals optimized their payoff by evolving a high response bias towards the signal response. Large cooperative groups did, however, not evolve a bias under high asymmetric costs, because the influence of even small biases amplify rapidly in large groups undermining the collective payoff. Interestingly, adding competition leads individuals to evolve stronger biases to avoid the costly error at the expense of providing less accurate information to others. Moreover, individuals in competitive groups wait for more evidence, further reducing the group’s average performance.

## Materials and methods

### The social DDM

In the social DDM, a group of individuals faces a binary decision task (signal present or absent), with four possible decision outcomes: Individuals can correctly decide that a signal is present, correctly decide that a signal is absent, incorrectly decide that a signal is present, or incorrectly decide that a signal is absent. The model is formalized as a drift–diffusion process where individuals continuously gather noisy evidence which updates their belief about the likelihood of the signal being present or absent in a Bayesian manner. For binary decisions, the evidence state—here denoted by *L*(*t*)—is typically formalized as the logarithm of the likelihood ratio of the signal being present versus absent given the gathered evidence at time point *t* [27, 40]. We assume that individuals first independently accumulate personal information about the state of the world, reflecting any experience prior to the second (i.e, social) phase. In a second phase, individuals make a decision. They do not receive any personal information anymore but they can gather social information (i.e., the choices of others).

In the first phase, individuals accumulate personal information [27, 41], whereby individuals start with a start point bias *z*_*p*_, which describes an initial preference for the signal or no signal option. Such start point biases allow individuals to account for asymmetries of the choice alternatives, for example, asymmetries in base rate (e.g. signal more frequent than no signal), or error costs (e.g., a miss being more costly than a FA; [5, 40]). Over time, individuals gather on average correct evidence described by a drift rate *δ*_*p*_ towards the correct option. The total amount of evidence *L*(*t*_*p*_) gathered until the end of the personal phase at time point *t*_*p*_ is described by a normal distribution with a mean of

$$E[L(t_{p})]=\{\begin{array}{ll} z_{p}+\delta_{p}\times t_{p}, & \text{if}\;\text{signal}\;\text{is}\;\text{present}\\ z_{p}-\delta_{p}\times t_{p}, & \text{if}\;\text{signal}\;\text{is}\;\text{absent} \end{array}$$ (1)

and a variance of

$$\begin{array}{c} Var\left[L\left(t_{p}\right)\right]=\sigma_{p}^{2}\times t_{p}, \end{array}$$ (2)

with *σ*_*p*_ being the diffusion rate (*σ*_*p*_ and *t*_*p*_ are set to 1 for simplicity; see S1 Fig for sensitivity analysis). The parameters *z*_*p*_ and *δ*_*p*_ change the evidence state in distinct ways: A positive (negative) *z*_*p*_ shifts the mean towards the decision boundary of the signal (no signal) option and is related to the decision criterion in signal detection theory; a positive (negative) *δ*_*p*_ shifts the mean towards the decision boundary of the correct (wrong) decision (Fig 1). The diffusion rate (*σ*_*p*_) describes the amount of random information (i.e., noise) and influences the variance of personal information. The amount of time *t*_*p*_ scales the mean and variance by influencing how long personal information is integrated.

Next, individuals enter a social phase also formalized as a drift–diffusion process. In this phase, individuals no longer sample personal information from the environment; instead, they can update their evidence based on the decisions of others. During this social phase, the evidence *L*(*t*_*s*_) changes continuously over time *t*_*s*_ until a decision is made (i.e., the evidence level exceeds either of the decision boundaries). In between the start of the social phase and the moment an individual makes a decision (*t*_*s*_), they can observe the choices of others. The incorporation of social information is described by the social drift rate which changes as a function of the majority of individuals who already decided. This Wiener process is approximated with a biased random walk with small discrete time steps Δ*t*_*s*_:

$$\begin{array}{c} L\left(t_{s}+\Delta t_{s}\right)=L\left(t_{s}\right)+\delta_{s}\left(t_{s}\right)\times \Delta t_{s}+\sqrt{\Delta t_{s}}\times \epsilon , \end{array}$$ (3)

with *δ*_*s*_(*t*_*s*_) representing the social drift rate and *ε* representing Gaussian white noise with a mean of 0 and a variance of 1 (changing the variance rescales the other parameters and does not change the model prediction; see [8]). The social drift rate *δ*_*s*_(*t*_*s*_) describes the change in an individual’s drift rate depending on the decisions of others (i.e., the impact of social information) and is modeled proportionally to the majority size *M*(*t*_*s*_) of individuals that already decided at time point *t*_*s*_ (e.g., [20, 25, 42, 43]):

$$\begin{array}{c} M\left(t_{s}\right)=N^{+}\left(t_{s}\right)-N^{-}\left(t_{s}\right), \end{array}$$ (4)

$$\begin{array}{c} \delta_{s}\left(t_{s}\right)=s\times M\left(t_{s}\right), \end{array}$$ (5)

where *N*^+^(*t*_*s*_) and *N*^−^(*t*_*s*_) are the number of individuals that decided the signal was present or absent, respectively, at time point *t*_*s*_, and *s* is scaling the strength of the social drift. Note that each choice added to a majority (or minority) has the same additive effect which is scaled by the parameter *s*. The amount of evidence an individual accumulates before making a decision is determined by the boundary separation *θ*. It describes the distance between the upper (signal) and lower (no signal) decision boundary, with the decision boundaries set at $\pm \frac{\theta }{2}$. Our model directly links individuals’ established cognitive processes to the unfolding collective dynamics. Note that the separation into two distinct phases allows a better interpretation of the agents’ behaviour as personal and social information accumulation are not convoluted [25]. Thus, boundary separation describes an individual’s willingness to wait for social information only. This separation thus exemplifies situations in which individuals have collected personal information prior to coming together in a social context, and exchange information (e.g., migrating animal groups or human group discussions). Further, it ensures that individuals with strong personal information will, on average, start closer to one of the decision boundaries and thus make faster decisions, as observed in groups across many biological systems [22–24, 44]. These fast decisions can, in turn, influence the drift rate of undecided individuals, thus capturing the natural dynamic of information flow from highly informed to lowly informed individuals [25]. See S2 Fig for the results of a scenario where additional personal information is gathered during the social phase, reducing an individual’s ability to time their decision according to information quality.

### The evolutionary algorithm

While some model parameters are not fully under an individual’s control (e.g., amount of personal information available), other behavioral parameters can be adaptively adjusted, including start point bias, social information use, and the amount of evidence needed to make a decision. To determine the adaptive behavioral parameters we embedded the social DDM into an evolutionary algorithm. Evolutionary algorithms allow fitness-maximizing parameter settings to jointly evolve by exposing them to selection pressure and mutation [38], making them highly suitable for game-theoretic problems, where the optimal behavior of individuals depends on the behavior of others. Individuals repeatedly went through three phases: (i) a simulation phase in which individuals repeatedly (on average 10 times) made decisions in the social DDM framework, (ii) an evaluation phase where the best individuals were selected and a new generation was build, and (iii) a mutation phase in which parameter (or trait) diversity of the new generation was ensured. This cycle was repeated a 1,000 times. At the start, we populated the simulations with 1.000 individuals. Each individual had three evolving traits: start point bias *z*_*p*_, boundary separation *θ*, and strength of the social drift *s* (Table 1). The parameters covered a wide range, ensuring the best solutions were included (range for start point bias: -0.5–2; boundary separation: 0.01–12; strength of social drift: 0–2). To ensure that the endpoints of the simulations were independent of their starting conditions of the population, we sampled the individuals’ initial parameters from a beta distribution with the minimum and maximum scaled to the respective evaluated parameter range, whereby the population mean of the beta distribution was sampled from a uniform distribution of the central 80% of the parameter range (e.g., 0.2–1.8 for the strength of social drift). Accordingly, we set the shape parameter *a* of the beta distribution to 1 and the parameter *b* to get a required mean (*init*) with $b=\frac{a}{init}-a$. In each generation, individuals were randomly and repeatedly (on average 10 times) sampled from the population to perform the social DDM simulation. When an individual was sampled, the likelihood to be sampled again was reduced by a factor five, ensuring that individuals were roughly equally often sampled. Each sampled individual—with their associated traits—performed the social DDM simulation as described above together with other sampled individuals (in different-sized groups; see below). After these simulations, individuals produced offspring based on their sum payoff, implemented via tournament selection: Three individuals were randomly sampled from the population and the individual with the highest payoff passed its traits to the next generation (results do not change when sampling more than three individuals). Finally, the traits of the new generation were exposed to mutation and crossover to ensure variation. Crossover was implemented by swapping two traits between a focal and a randomly drawn individual with a probability of 0.05. For the mutation process, we added Gaussian noise to a trait with a mutation probability of 0.02 and a standard deviation of 5% of the evaluated parameter ranges. These procedures were repeated for 1,000 generations, which ensured that populations converged to stable endpoints. We measured the evolved parameters by averaging the parameter values of the last 10 generations across eight populations.

**Table 1:** Description of the model parameters. Underlined parameters evolve in the evolutionary algorithm.

Model feature	Parameter	Description
Start point in social phase	$L\left(t_{p}\right)∼N\left(\underline{z_{p}}\pm \delta_{p},\sigma_{p}\right)$	Parameters influencing the evidence gathered during the personal phase *L*(*t*_*p*_), which then served as the start point in the social phase. *δ*_*p*_, $\underline{z_{p}}$ determine the mean and *σ*_*p*_ the variance of a normal distribution (see Fig 1). A positive (negative) *δ*_*p*_ shifts the mean towards the correct (wrong) option, reflecting the amount of correct evidence gathered. A positive (negative) start point bias $\underline{z_{p}}$ shifts the mean towards the signal (no signal) response and allows the decision maker to account for error cost asymmetries.
Boundary separation	$\underline{\theta }$	The boundary separation determines the distance between the decision boundary for signal and no signal, thereby influencing how much evidence an individual accumulates before making a decision (red horizontal lines in Fig 1). Increasing the boundary separation increases the potential for social information use.
Social drift rate	$\delta_{s}\left(t_{s}\right)=\underline{s}\times M\left(t_{s}\right)$	*δ*_*s*_(*t*_*s*_) describes the incorporation of social information with $\underline{s}$ regulating its strength by scaling the influence of the majority size (of the individuals who already decided for a particular option *M*(*t*_*s*_)) on the social drift rate (see Fig 1).

We systematically varied three features: group size, error cost asymmetry, and payoff level, to study their impact on the evolved parameter traits. First, we varied the group size (1, 5, 10, 20, and 50) in which individuals made decisions. Second, we varied error cost asymmetry to investigate how individuals in groups should account for asymmetric error costs. Under symmetric costs, an individual received one unit of payoff for a correct decision (hit or correct rejection) and lost one unit for a wrong decision (miss or false alarm). We modeled asymmetries in error costs by increasing the cost ratio of a miss compared to that of a false alarm, reflecting that missing a signal (e.g., a predator or disease) is generally more costly than a false alarm. Across the three cost scenarios, the average error cost was kept constant at 1 unit but we varied the miss/false alarm cost ratio (1, 2, and 4). The cost of a miss was set at either 1, $\frac{4}{3}$, or $\frac{8}{5}$, and the cost of a false alarm at 1, $\frac{2}{3}$, or $\frac{2}{5}$. The average cost and benefits are thus constant across cost conditions. The cost ratio scheme reflected cost schemes used in previous studies on signal detection problems (e.g., [5, 7, 45]). We kept the base rate (i.e., overall probability of signal to occur) constant at 50% (see S3 Fig for a sensitivity analysis with varying base rates). Moreover, we added a time cost of 0.05 units per second (see S4 Fig for a sensitivity analysis with varying time cost, and S5 Fig for an analysis of time cost asymmetries). This time cost reflects the commonplace benefit of making fast choices [29]; it also means that at a certain point in time, the benefit of a correct choice no longer outweighs the time cost. Third, we varied the payoff level: Individuals received a payoff based on either the mean group payoff (cooperative scenario) or their own payoff (competitive scenario). Individuals in the cooperative scenario were selected to maximize their group performance and individuals in the competitive scenario to perform better than others.

After running the evolutionary algorithm we inspected the population trajectories. In all scenarios, the populations converged to a single equilibrium, independent of the starting conditions. This indicates that the algorithm found a single robust solution for each combination of group size, error cost asymmetry, and payoff level. Thus, we did not find stable co-existences of different strategies. S6 Fig shows the trajectories of the three evolving parameters for randomly sampled exemplary scenarios, illustrating the convergence. S7 Fig shows example runs of the social DDM simulations for each of the 24 conditions, using the parameters of the evolutionary endpoints of the specific setting, to illustrate the temporal unfolding of the decisions in a social context.

### Performance evaluation

To gain a deeper understanding of individuals’ behavior at the evolutionary endpoints, we performed additional social DDM analyses with fixed parameter settings (i.e., no evolution of parameters). To investigate the effect of the start point bias *z*_*p*_, boundary separation *θ*, and strength of social drift *s*, on individuals’ performance (i.e., their payoff and their hit and correct rejection rates), we varied the parameter of interest—for different group sizes and error costs—while fixing the other two parameters at their evolved level of cooperative groups, and measured individuals’ performance over 1,000,000 repetitions.

### Evaluating the influence of competition

Because the endpoints of cooperative and competitive groups differed (see Results), we studied how competition drives populations away from the optimal behavior of cooperative groups. We again varied the start point bias *z*_*p*_, boundary separation *θ*, or strength of social drift *s* while fixing the remaining two parameters at their evolved level of cooperative groups. We also introduced interindividual heterogeneity by assigning half of the individuals of each group a higher parameter value, and the other half a lower value, splitting groups of five randomly (difference in start point bias: 0.2; boundary separation: 0.4; strength of social drift: 0.1). This allowed us to measure the benefits of having a higher (or lower) parameter value than the other group members and, thereby, the effect of competition. We measured payoffs over 1,000,000 repetitions for each parameter combination. Competitive groups are expected to evolve parameters such that individuals do not gain a personal advantage by having higher or lower parameters. Note that this analysis only approximates the evolutionary endpoints of competitive groups because of a different implementation. In this evaluation, the group consists of two distinct behavioral types with two fixed parameters; in the evolutionary algorithm groups are heterogeneous across all parameters and the parameters can freely evolve in concert.

## Results

### Asymmetry in error costs

**Fig 2:**
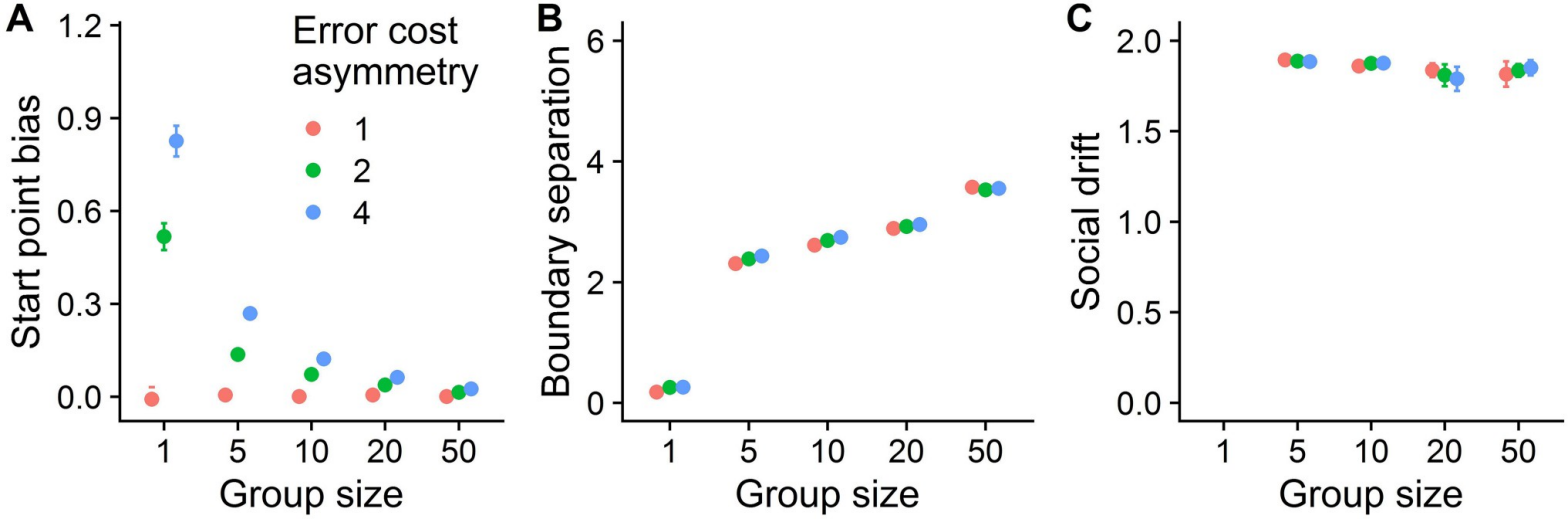
Outcomes of the evolutionary algorithms per group size and error cost in cooperative groups. (A) When costs are symmetrical (i.e., error cost asymmetry = 1), no start point bias evolves at any group size. With increasing cost asymmetry, small (but not large) groups evolve a larger bias. (B) Across all error costs, larger groups evolve a higher boundary separation. (C) Across all combinations of group size and error cost, a high social drift evolves. Dots and error bars represent the mean and standard deviation, respectively, across the eight evolved populations. For exemplary evolutionary trajectories see S6 Fig.

**Fig 3:**
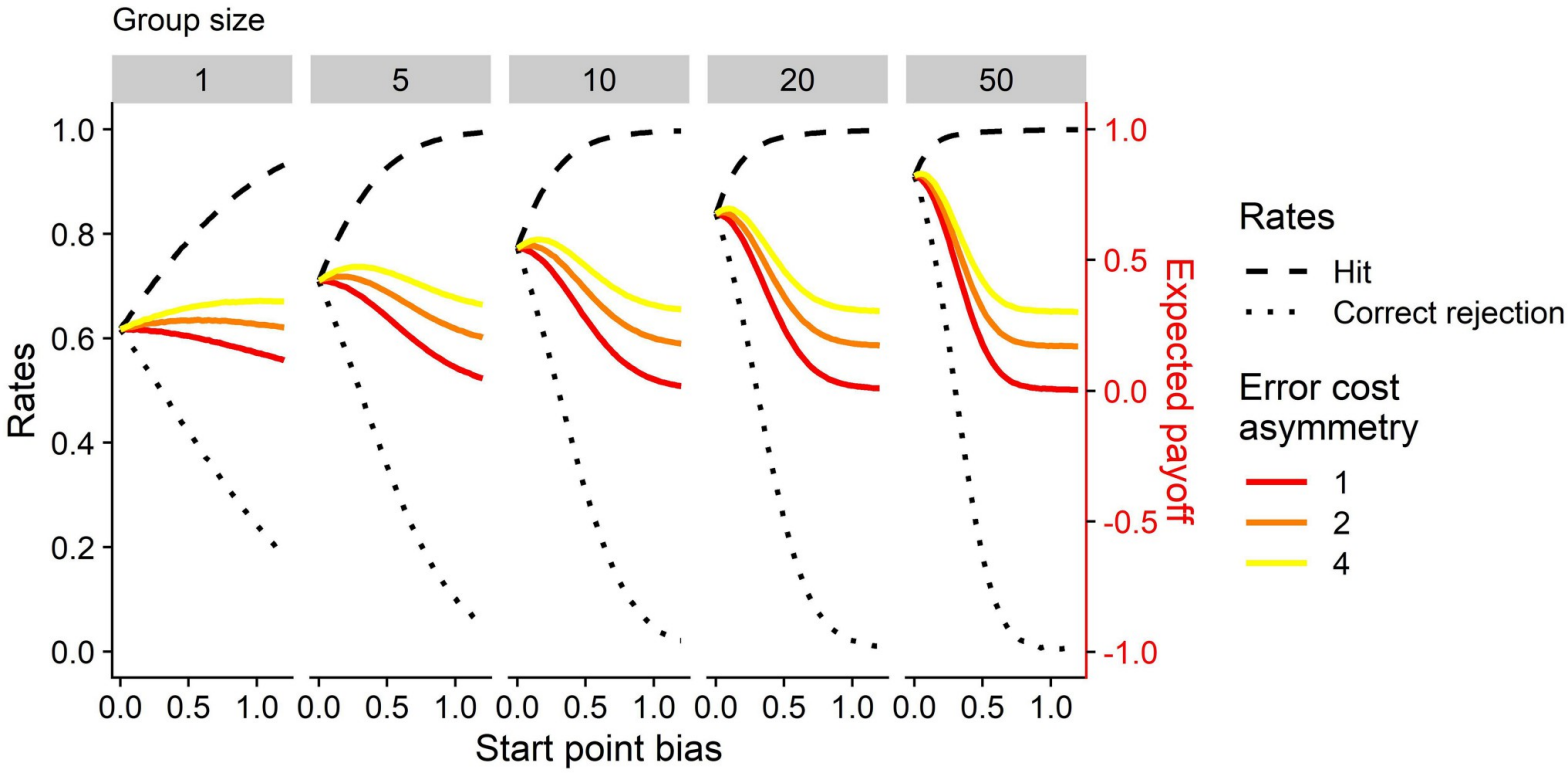
Hit and correct rejection rates (black lines, left axis) and payoff (colored lines, right axis) as a function of start point bias for different group sizes and error costs for cooperative groups. Across all group sizes, increasing the start point bias towards the decision boundary of the signal leads to an increase in the hit rate, but simultaneously to a decrease of the correct rejection rate. Under symmetrical error costs, individuals across all group sizes maximize their payoff by maximizing the hit and correct rejection rate alike; this occurs at a bias close to 0. Under asymmetric costs, individuals need to ensure a high hit rate in order to avoid costly misses. Small groups achieve this by developing a bias. Large groups achieve a high hit (and correct rejection) rate without a start point bias, and therefore maximize the payoff at a much lower bias. The boundary separation and strength of the social drift were fixed at the endpoints of the evolutionary algorithms for each combination of group size and error cost.

**Fig 4:**
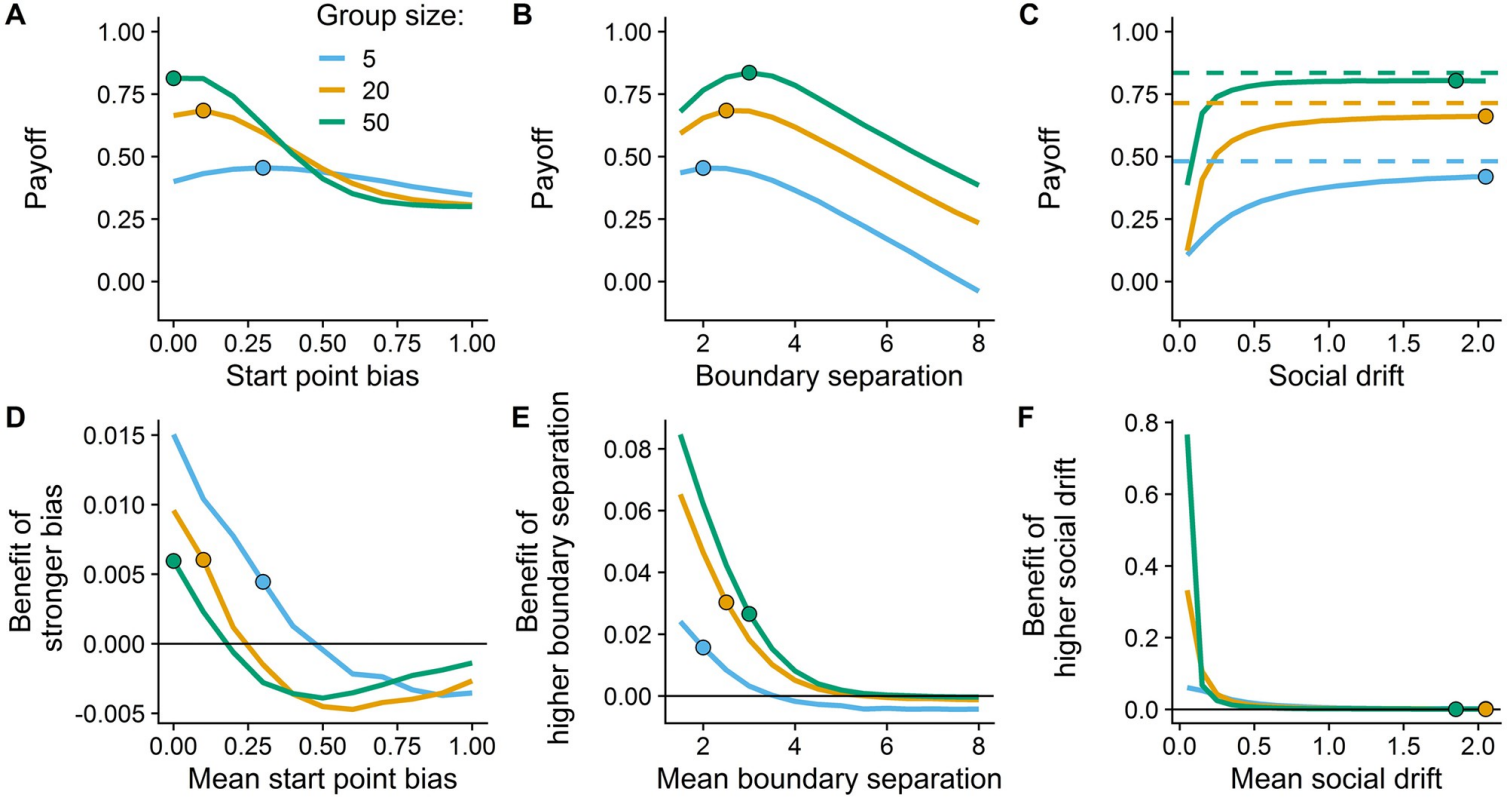
Payoff analysis with varying start point bias, boundary separation or strength of the social drift. (A–C) The mean payoff of individuals in different-sized, cooperative groups across the three key parameters under high asymmetric error costs (cost asymmetry: 4). In these simulations, one evolved parameter was varied (x-axis), while the other two were fixed at their evolved level of cooperative groups. Larger groups maximized their payoffs (indicated by dots) at (A) a lower start point bias and (B) higher boundary separation compared to small groups. (C) All group sizes maximized their payoff at the highest level of social drift strength. Dashed horizontal lines show the mean payoff of the first responder. With increasing strength of the social drift, the mean payoff of all group members approximated the payoff of the first responder. (D–F) The benefits of individuals in competitive groups having above-average values in the three key parameters under high asymmetric error costs. Positive (negative) y-values indicate that individuals with above-average (below-average) values in the respective parameter achieved a higher payoff. Competitive groups evolved parameter values at which their members did not profit from having a higher (or lower) parameter value (i.e, where colored lines meet the solid horizontal line at zero), which approximates the outcomes of the evolutionary algorithm. These values partly differed from optimal outcomes in cooperative groups (dots), indicating a social dilemma. At these evolved endpoints of the cooperative groups individuals in competitive groups benefited from having a higher (D) start point bias and (E) boundary separation. (F) Cooperative and competitive groups did not differ in their evolved value of social drift.

Fig 2 shows the evolved parameters for different group sizes and error costs in cooperative groups. When errors costs were symmetrical, no start point bias developed in cooperative groups of any size (Fig 2A). With increasing cost asymmetry, individuals acting alone and in small cooperative groups evolved a bias towards the signal decision boundary avoiding the costly error. Large cooperative groups, however, did not evolve a bias even under high asymmetric error costs. An explanation for the lack of bias in large cooperative groups can be found in Fig 3, which shows the hit and correct rejection rates, as well as the payoffs, for different group sizes and biases (while fixing the boundary separation and the strength of the social drift at the evolved level of the specific group size; see Fig 2). Across all group sizes, increasing the start point bias increased the hit rate but decreased the correct rejection rate. Under symmetrical costs, the highest payoffs were obtained at a bias level of 0, which maximizes the combined sum of the hit and correct rejection rate. However, when misses became more costly than false alarms, single individuals and small groups maximized their payoff at a relatively high bias to avoid costly misses. In large groups, by contrast, a bias close to 0 was optimal. Large groups achieved high hit and correct rejection rates without a start point bias, and were very sensitive to small biases. Increasing their start point bias did increase their hit rate, but this did not outweigh the associated costs of the steep drop in the correct rejection rate. The steepness of this drop increased with group size. In other words, a strong bias in large groups would lead to many false alarms; this can be avoided by reducing the bias. This is further illustrated in Fig 4A, which directly compares the performance of different sized groups across different bias levels at the highest level of error cost asymmetry. Large groups were sensitive to biases: They outperformed small groups in the absence of a starting point bias, but were outperformed by small groups under high levels of start point bias.

### Boundary separation

Fig 2B shows that individuals in larger groups evolved a larger boundary separation, implying that they required more evidence to trigger a decision. This effect was independent of asymmetry in error costs. Fig 4B directly compares the payoffs of different-sized groups for different levels of boundary separation at the highest level of error cost asymmetry, further confirming the benefits of evolving higher boundary separations for larger groups. Individuals need more information in larger groups because the potential benefits of social information are higher in larger groups; the relative benefits of waiting longer for social information should therefore also be higher. The benefits of waiting for social information were influenced by the time costs: the higher the time costs the lower the amount of evidence individuals required to make a decision (S4 Fig).

### Social drift

Across all group sizes and error costs, the strength of the social drift evolved to the maximum level (Fig 2C). The evolution towards the maximum value indicates the effectiveness of a simple “copy-the-first” heuristic, whereby individuals are very likely to imitate the decision of the first responders via a strong social drift (see S7 Fig for exemplary simulations). This simple heuristic performs so well because of the way personal information is gathered. Individuals with more accurate personal information start, on average, closer to a decision boundary than individuals with less accurate information. This gives rise to a process of self-organization, with more accurate individuals making faster decisions [25]. The first responders therefore generally achieve a higher payoff compared to later-deciding individuals, independent of strength of the social drift or group size (indicated by the dashed lines being higher than the solid lines in Fig 4C). For later deciding individuals, the best strategy is thus to increase the social drift to the maximum level in order to imitate the first decisions, thereby saving costly time. We set the maximum strength of the social drift to two. Allowing a higher maximum level would have a minor effect as indicated by the saturation of the curves at s = 2 in Fig 4C.

### Competitive versus cooperative groups

**Fig 5:**
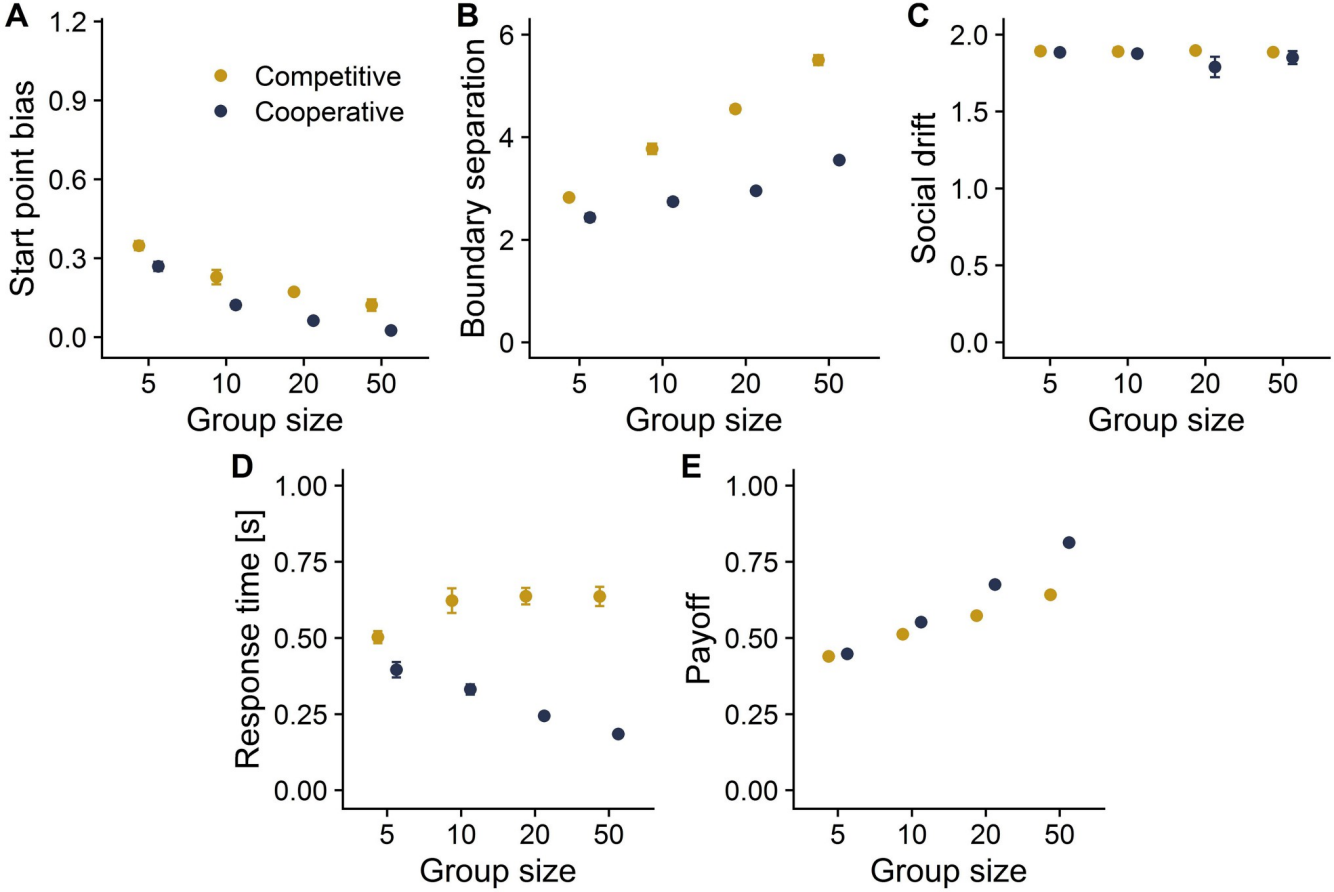
Evolutionary outcomes of cooperative and competitive groups at an error cost ratio of 4. Across all group sizes, competitive groups evolved (A) a larger start point bias and (B) larger boundary separation, indicating a conflict between individual- and group-level interests. (C) Both cooperative and competitive groups evolved the maximum strength of the social drift. (D) Cooperative groups made, on average, faster choices than competitive groups, and this difference increased with group size. (E) At large, but not small, group sizes, cooperative groups outperformed competitive groups. Dots and error bars represent the mean and standard deviation of the endpoints of the evolutionary simulations, respectively.

Next, we investigate how the evolved strategies in cooperative groups—with individuals selected to maximize the group payoff—differ to the evolved strategies in competitive groups—with individuals selected to maximize their own payoff. Across all group sizes, competitive groups developed a stronger start point bias towards the signal boundary than did cooperative groups (Fig 5A). To investigate this result, we introduced interindividual heterogeneity in the bias level within competitive groups and compared the payoffs of these different bias levels. When the average start point bias was set to a level that maximized the mean group payoff (dots in Fig 4A and 4D), competitive individuals with a higher start point bias gained higher individual payoffs, and this advantage only disappeared when the group had a substantially higher mean bias (Fig 4D). This could be due to the tension between providing good information to group members and maximizing one’s own payoff. Reducing their start point bias enables individuals to provide more accurate social information, while increasing their start point bias helps them avoid the high personal costs of a miss—but this comes at the expense of accuracy and therefore results in more misleading social information.

For all group sizes, competitive groups evolved higher boundary separations than did cooperative groups (Fig 5B). Fig 4E shows that, at the maximum payoff level of cooperative groups (dots), competitive individuals benefited from having a slightly higher boundary separation. Only at substantially higher levels of mean group boundary separation did this benefit disappear, thus driving the boundary separation in competitive groups to higher values.

Both cooperative and competitive groups evolved to the maximum level of social drift strength (Fig 5C). In line with this, individuals with a lower social drift strength never outperformed individuals with a higher social drift strength (indicated by the strictly positive values in Fig 4F). This suggests strong benefits of using social information, or even copying the first responder, independent of group size or cooperative setting.

Finally, we investigated how competition influences the evolved behaviour. We found that individuals in cooperative groups made, on average, faster choices and achieved a higher payoff than individuals in competitive groups (Fig 5D and 5E). Cooperative groups made faster choices with increasing group size, whereas competitive groups took slightly longer with increasing group size (Fig 5D). Although both cooperative and competitive groups achieved a higher payoff at larger group sizes, cooperative groups benefited much more from larger groups (Fig 5E). This is because the larger start point bias and boundary separation that evolved in competitive groups partly undermined the benefits of collective decision making. As a sensitivity analysis, we repeated the simulations with varying time costs and distribution characteristics of personal information, replicating the main findings (see S1–S4 Figs).

## Discussion

We investigated the evolution of individuals’ adaptive decision rules across different group sizes, error cost asymmetries, and competitiveness. When the cost of a miss was higher than the cost of a false alarm, individuals in small groups evolved a start point bias to avoid costly misses. This corroborates earlier findings showing that individuals faced with asymmetric error costs shift their decision criterion (in a signal detection theory analysis; [7]) or their start point bias (in a DDM framework; [5, 45]) to avoid the more costly error. Strong start point biases are adaptive in small groups, but in large groups they amplify quickly, typically leading groups to decide for signal. This results in a high hit rate, but also a high false alarm rate; larger collectives may therefore suffer when individuals do not adjust their biases accordingly.

Such individual response biases may have important implications for collective systems such as crowds. If individuals adjust their response bias to avoid the more costly error, their adjustment must be tuned to the expected group size. While large adjustments might be wise in a small group, such large adjustments can quickly escalate in large crowds, which are more vulnerable to false alarms. For instance, after terrorist attacks like 9/11 in 2001 or the Paris attacks in 2015, some individuals may have adjusted their response bias towards an alarm response. Such adjustments might play a role in increases of deadly panics in large crowds after terroristic events. In post-9/11 Chicago, for example, several club visitors mistook pepper spray for a poison gas attack; the resulting panic left 21 dead [46]. The risk of amplification might be further worsened in competitive groups, which evolve a higher bias than cooperative groups. When individuals aim to maximize their own payoff, they are willing to accept a higher level of false alarms at the expense of the collective well-being. In our model, large competitive groups performed substantially worse than large cooperative groups, partly due to a higher evolved start point bias. In other words, competition comes with a price to the whole group as it reduces the efficiency of social information exchange (see also ‘Price of Anarchy’: [47]). In high-stress situations (e.g., a perceived terrorist attack) people’s behavior often shifts from cooperative to competitive [48, 49]. Taken together, our results highlight a social dilemma and the potential danger posed to groups by individuals with a high response bias. Future research should aim to provide a more detailed understanding of how information spreads in such situations.

Groups are particularly vulnerable to information cascades due to their high reliance on social information [20]. We found that the strength of the social drift evolves to a maximum across group size, cost asymmetry, and competitiveness, indicating a reliance on a copy-the-first strategy. This finding confirms previous studies on strategic delay, which describe a Nash equilibrium in which everyone initially delays their choice [50, 51]. As time passes, the individual with the best information can assume that, since no-one else has made a decision, their information is better than that of other group members. Because the individual with the best information is expected to decide first, others then simply imitate this decision. Adopting a copy-the-first strategy allows individuals to rely on the social source with the strongest evidence; it also saves time, since individuals do not need to wait for others to decide.

Crucially, these studies—and ours—assume that accurate choices are made faster than inaccurate choices, which is often observed empirically [25, 52]. We implemented this accuracy-response time correlation by allowing individuals to gather personal information prior to the social phase. Consequently, the start point during the social phase was, on average, closer to the correct decision boundary. Alternatively, researchers assumed collapsing boundaries or varying drift rates to explain such patterns [52]. When correct choices are not made faster and the first responders do not provide above-average information quality, lower levels of social drift can be adaptive under some conditions (see S2 Fig). Furthermore, previous studies assumed that individuals have a similar speed–accuracy trade-off (e.g., the same boundary separation; [29]). If individuals differ in their speed–accuracy trade-off [53], the positive association between first responder and information quality will attenuate. In this scenario, the first responder is more likely to emphasize speed, at the expense of accuracy. When individuals differ in their speed–accuracy trade-off, favoring the decisions of individuals who respond quickly might thus undermine the benefits of a copy-the-first strategy. Individual differences in speed–accuracy trade-offs and inefficient coordination of decision time and accuracy could explain why former empirical studies studying sequential decision making in real time, have not found that individuals copy the first decision maker [11, 23, 25]. Future research could investigate how individual heterogeneity in speed–accuracy trade-offs undermines the presence of fast and accurate choices and their consequences for the collective.

Another interesting extension of our framework is the evolution of more complex strategies when integrating social information. We assumed a linear relationship between majority size and drift rate to minimize the number of free parameters and improve the interpretability of the results. Although such simple strategies are commonly assumed (e.g., [20, 42, 43]), a diverse set of more complex responses to social information have been described, such as quorum thresholds [4, 54–57]. Quorum thresholds initially downweight small minorities, before ramping up social information use once the majority reaches a critical threshold. Other possible strategies include the use of the relative difference in the numbers of individuals favoring each option [58, 59], the incorporation of decision order (e.g. by up-weighting recent choices [60, 61], the consideration of conflicting preferences [62], or the incorporation of social information by a one-time update in the evidence [34, 35]. The exact strategy of social information integration can influence the temporal dynamic of the collective process. For example, groups using quorum thresholds are expected to have an initial phase with few sporadic choices until the quorum is reached and the undecided individuals follow the majority option within a short amount of time [56]. Groups integrating social information by a one-time update of the evidence are expected to decide in a round-based manner (i.e., in short waves) instead of making choices continuously over time [34, 35]. The DDM framework not only accounts for choices but also their response times. The time it takes for an individual to make a choice can, therefore, convey important information. Individuals, for example, can infer the accuracy of choices from their response times [63]. Karamched et al., showed that even non-decisions can convey information if one choice alternative is, on average, selected faster [35]. Future work unraveling the dynamics underlying sequential choice, could thus benefit from taking a mechanistic approach to the dynamics of social updating, to understand the temporal dynamics of such systems in more detail.

Next to the process of social updating, the process of social information transmission is also likely to play a key role. The social information in sequentially deciding groups—as studied here—is signalled by a one-time choice of the individual. Other influential models of collectives assume that individuals continuously have access to the current belief or opinion of the connected group members such as the Vicsek model [64] or Voter model [65]. Biased (or informed) individuals in this kind of interaction process establish their influence via a continues pull force which determines how they contribute to the collective outcome. For example, in these models the influence of strongly biased (or informed) individuals on the outcome is predicted to be strongly mediated by the presence of unbiased (or uninformed) individuals [66, 67]. Whether uninformed individuals promote or undermine the influence of early deciding strongly biased individuals in groups of sequentially deciding individuals would be another interesting future investigation.

The willingness to wait for social information—described by boundary separation—also influences collective dynamics. We found that boundary separation increased with group size, meaning that individuals in larger groups required more evidence to make a decision. This increase was, however, relatively small in cooperative groups (Fig 5B), and cooperative groups made even faster choices at larger group sizes. This can be explained by larger groups also being more likely to contain strongly informed individuals (e.g., the many eyes principle; [68]). Similar mechanisms have been described in shoaling fish, which have been shown to make faster (and more accurate) choices in larger groups [43]. However, in competitive groups, individuals profited from requiring even more evidence at the expense of decision speed and group performance. This result shows similarities with a well-known finding in social psychology first demonstrated by [69]: the bystander effect. According to the bystander effect, people are less likely to offer help the more other people are present. We show that waiting longer to see whether others respond can be an adaptive strategy, as individuals in larger groups should only make a choice (e.g., whether to offer help) with strong evidence. Matching this prediction, a study using CCTV footage found that increased bystander presence reduced individuals’ likelihood of intervening (e.g., via increased boundary separations) while simultaneously increasing the likelihood of someone intervening [70]. The bystander effect could be explained as a rational adaptation to maximize informational gain in competitive groups with varying group sizes. However, future work should investigate this further as previous work on the social dynamics in situations less fraught with moral connotations did not find a larger boundary separation in larger groups [25].

Although—as discussed above—some aspects of the evolved behaviours resemble real behaviour others seem to differ. We, therefore, emphasize that animal and human cognition is shaped by not accounted evolutionary processes and constraints. Thus, animals and humans typically only approximate normative predictions. Such predictions are, however, insightful as they shed light on the game-theoretical problems collectives are facing, how they could resolve them and what consequences they impair if they fail.

## Conclusion

To conclude, in the presence of asymmetric error costs, individuals adjust their response bias to the group size in order to maximize their payoff. In particular, individuals in large groups should avoid strong start point biases, which would frequently trigger false information cascades. Further, individuals face a social dilemma: The indifference of competitive individuals to the negative consequences of their response bias and their tendency to wait for more social information leads groups—especially large groups—to fail to reap the collective benefits of making collective decisions. In the real world, asymmetric costs are the rule rather than the exception; our results therefore might have important implications for understanding a wide range of social dynamics, including police officers’ decisions to shoot, crowd panics, and escape responses under predation risk.

## Supporting information

S1 FigSensitivity analysis showing the outcomes of the evolutionary algorithm for different distributions of personal information in competitive and cooperative groups at an error cost ratio of 4.Left (right) panels show very low (high) mean start points *δ*_*p*_; upper (lower) panels show very small (large) variance $\sigma_{p}^{2}$ (both indicated in gray). Central panels show the personal information distribution used in the main analysis. The results approximate the main results for a wide range of characteristics of the personal information distribution. For a few conditions populations did not evolve to a single equilibrium, as evidenced by the broad error bars. This is further investigated in S8 Fig. Dots and error bars represent the mean and standard deviation across the eight populations.(TIFF)

S2 Fig The outcomes of the evolutionary algorithm if both personal and social information are aggregated during the second phase. Results are shown for different personal drift rates with a highly asymmetric error cost. Note that the first phase is the same as described in the main text. The results approximate the main results for most parameters and personal drift rates. However, large cooperative groups with high personal drifts evolve lower social drift rates. This is because accuracy-response time correlations (which allow individuals to coordinate their choice according to their information quality) originate from the start point being closer towards the correct option. If fast individuals are more accurate the ‘copy-the-firsts’ pays off. High personal drift causes choices to be more driven by the personal drift instead of start point, undermining the individuals ability to coordinate according to their information quality. In such cases, it is better for cooperative individuals in large groups to follow a slowly emerging majority. Note that, in contrast to the main setting, boundary separation in this implementation does not only describe the individuals’ willingness to wait for social information but additionally their preferred speed-accuracy trade-off. Dots and error bars represent the mean and standard deviation across the eight evolved populations.
(TIFF)

S3 Fig Sensitivity analysis showing the outcomes of the evolutionary algorithm for different base rates with the error costs being symmetrical. The overall probability of a signal being present varies from 50% (as in the main text) to 80% (i.e., the signal being 4 times as frequent). Individuals should adapt to varying base rates in a similar fashion as to varying cost asymmetries by adjusting their start point. In the presence of base rate asymmetries, they should adjust their start point towards the choice alternative which is a-priori more likely to be correct (here signal). If the signal was very likely to be present, competitive groups did not evolve a single equilibrium and some populations developed the simple strategy of always choosing signal. This is further investigated in S8 Fig. Dots and error bars represent the mean and standard deviation across the eight populations.
(TIFF)

S4 Fig Sensitivity analysis showing the outcomes of the evolutionary algorithm for different time costs and group sizes in competitive and cooperative groups at an error cost ratio of 4. Central panels show the time cost (indicated in gray) used in the main analysis. Upper (lower) panels show very small (large) time costs. Under very small and large time costs almost all main results were reproduced: (A) Small (but not large) groups evolved a start point bias, with competitive groups evolving a higher bias; (B) larger groups evolved a higher boundary separation, with competitive groups evolving a higher boundary separation; (C) groups evolved a high social drift strength, reflecting a copy-the-first heuristic; and (D) larger groups performed better, with cooperative groups outperforming competitive groups. Two scenarios deviated slightly from the main results. First, at very low time costs (0.01), large (50) cooperative groups did not evolve a social drift strength close to 2, but instead fluctuated around a value above 1. In large groups such social drift values already result in a high likelihood to copy first responders. Given the extremely low time costs, further increasing the drift does not improve the performance. Second, in large groups (50) facing high time costs (0.25), not all competitive populations converged to the same solution, as evidenced by the broad error bars. This is further investigated in S10 Fig. Dots and error bars represent the mean and standard deviation, respectively, across the eight populations.
(TIFF)

S5 Fig Sensitivity analysis showing the outcomes of the evolutionary algorithm without (upper panel) and with (lower panel) time costs asymmetries. To exemplify situations where time costs in one state of the world are much higher (i.e., responding fast when signal is present is much more important), we introduced time costs asymmetries by implementing time costs only if the signal was present. We doubled the time costs of the main analysis to keep the average time cost constant (i.e., we increased time costs from 0.05 to 0.1). The results with and without time costs asymmetries are similar indicating that it is not beneficial to adjust the start point to adapt to time cost asymmetries. If time costs are only present in one state of the world, agents still try to make fast choices as they are not aware of the state of the world they are in.
(TIFF)

S6 Fig Example trajectories of the evolutionary algorithm. Shown are the evolutionary trajectories of bias (left), boundary separation (center), and social drift strength (right), for six additional scenarios. These scenarios were randomly drawn from all 30 analysed scenarios. The corresponding parameter settings are shown in the right panels. Colored lines represent the average parameter value within each of the eight evolving populations; black lines indicate the average across all eight populations.
(TIFF)

S7 Fig Example simulations of the social DDM with the parameters obtained from the evolutionary endpoints. Shown are a single exemplary runs for each group size, error cost asymmetry and competition level with a 50% probability of the signal being present or absent. The red horizontal lines indicate the decision thresholds.
(TIFF)

S8 FigExamples of evolutionary algorithm simulations of scenarios shown in S1 Fig where populations did not converge to a single equilibrium.(A–D) In competitive groups (group size = 20) with low *δ*_*p*_ and intermediate $\sigma_{p}^{2}$ populations either converged to be highly biased, making rapid choices (i.e., low boundary separation; e.g., pink and purple lines) or to be less biased with larger boundary separation (green and light red lines). (E–H) Similarly, in competitive groups (group size = 20) with high $\sigma_{p}^{2}$ and intermediate *δ*_*p*_, populations converged either to highly biased groups with low boundary separation (e.g., brown and blue lines) or less biased groups with higher boundary separation (green and purple lines). For both examples of nonconvergence, reducing the start point bias (or increasing the boundary separation) in a highly biased population is likely to be disadvantageous. Such a strategy would likely result in similar choices, since the group members are likely to pull the individual towards the signal response, but at higher time costs because the response would be slightly delayed. Each line represents the average parameter value of one of the eight evolving populations.(TIFF)

S9 Fig Example of evolutionary algorithm simulations shown in S3 Fig where populations did not converge to a single equilibrium. If signal was present in 80% of the simulations, populations with large competitive groups (group size = 50) either converged to be highly biased and make rapid choices or to be less biased with a larger boundary separation. While populations with lower start point bias would, on average, perform better, reducing the start point bias (or increasing the boundary separation) in a highly biased population is likely to be disadvantageous. Less biased individuals would likely be pulled towards the signal response. These results mirror the behaviour found when providing individuals with low quality information (see also S2 and S8 Figs).
(TIFF)

S10 Fig In large (50) competitive groups under high time costs (0.25), populations converged to one of two equilibria. One equilibrium (blue, turquoise, green and light green lines) followed the pattern of our main result, namely (A) low start point bias, (B) medium boundary separation, (C) maximum social drift, and (D) high performance. In the other equilibrium (purple, pink, orange and brown lines), populations converged to a nonsocial behavior with individuals (A) being highly biased, (B) barely waiting, and (C) not incorporating social information, resulting in (D) low performance. This solution is stable, since a less biased individual with larger boundaries is likely to suffer from higher time cost by waiting for and ultimately following other individuals (who almost always choose ‘signal’). Each line represents the average parameter value of one of the eight evolving populations.
(TIFF)
