# Supplementary figures and images for: Avoiding costly mistakes in groups: The evolution of error management in collective decision making
Source: PLoS Comput Biol. 2022 Aug 19;18(8):e1010442. doi: 10.1371/journal.pcbi.1010442 (PMC9432742; doi:10.1371/journal.pcbi.1010442)

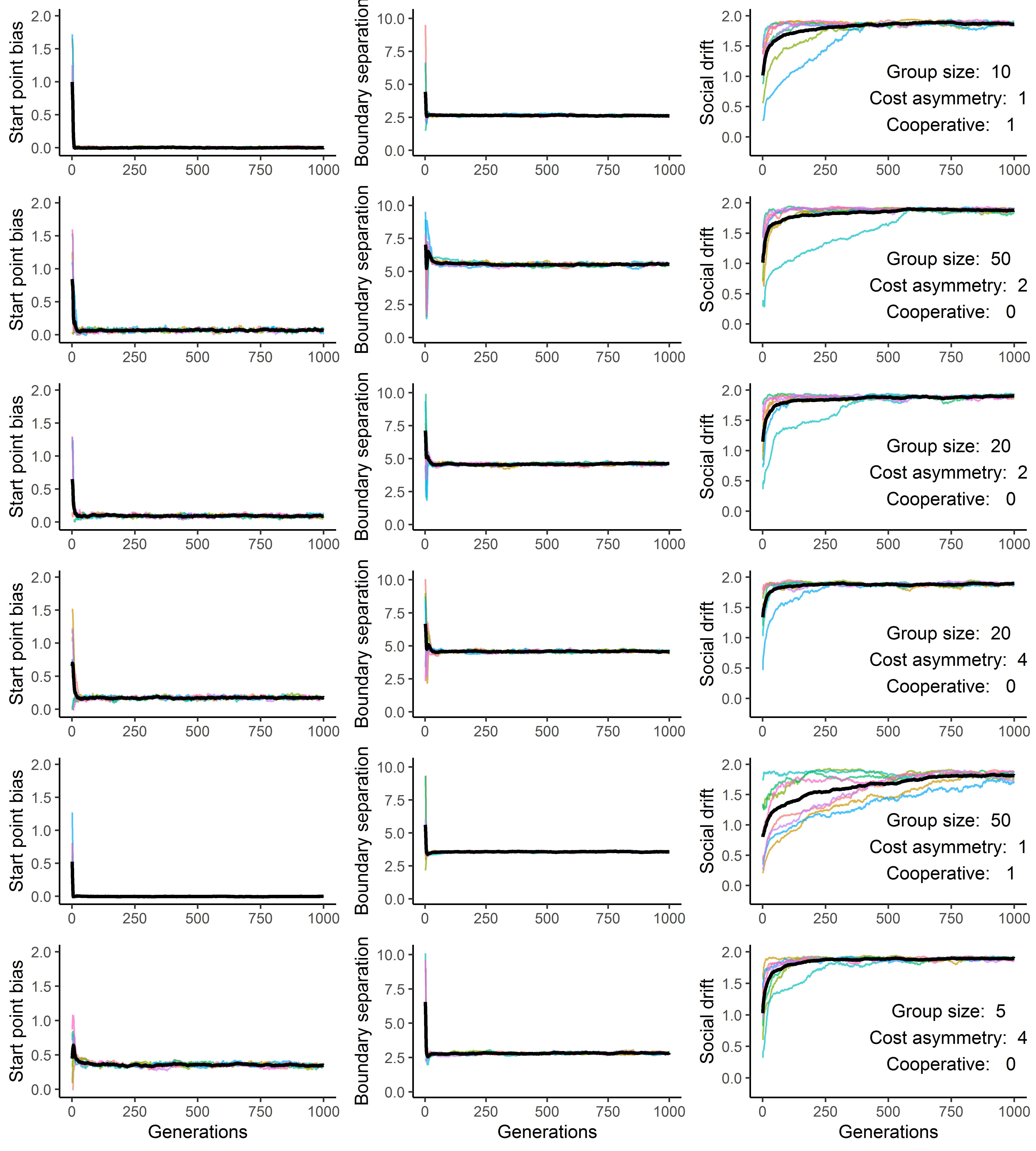

Supplement: S6 Fig — Shown are the evolutionary trajectories of bias (left), boundary separation (center), and social drift strength (right), for six additional scenarios. These scenarios were randomly drawn from all 30 analysed scenarios. The corresponding parameter settings are shown in the right panels. Colored lines represent the average parameter value within each of the eight evolving populations; black lines indicate the average across all eight populations. (TIFF) [file pcbi.1010442.s006.tiff]

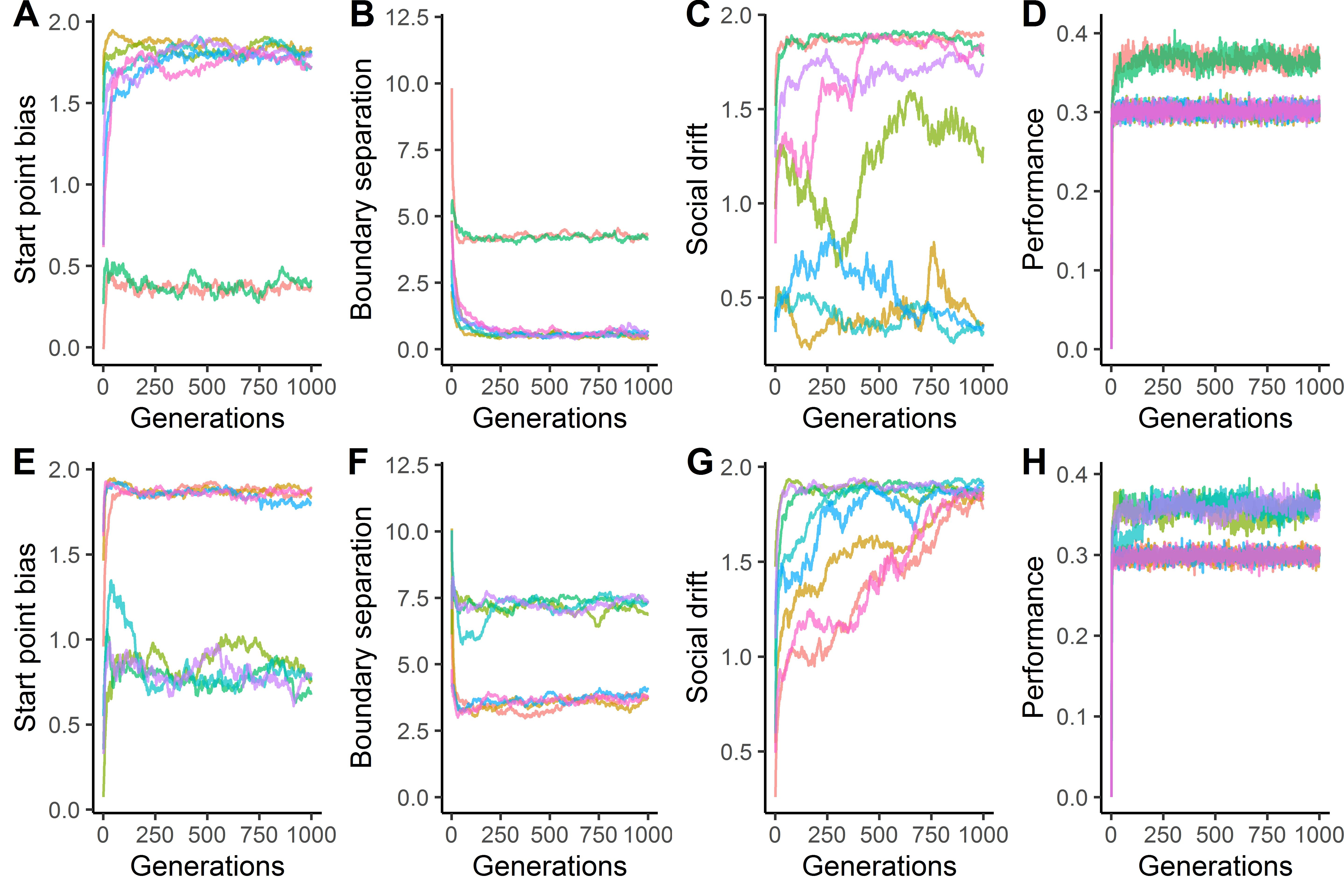

Supplement: S8 Fig — (A–D) In competitive groups (group size = 20) with low δp and intermediate σp2 populations either converged to be highly biased, making rapid choices (i.e., low boundary separation; e.g., pink and purple lines) or to be less biased with larger boundary separation (green and light red lines). (E–H) Similarly, in competitive groups (group size = 20) with high σp2 and intermediate δp, populations converged either to highly biased groups with low boundary separation (e.g., brown and blue lines) or less biased groups with higher boundary separation (green and purple lines). For both examples of nonconvergence, reducing the start point bias (or increasing the boundary separation) in a highly biased population is likely to be disadvantageous. Such a strategy would likely result in similar choices, since the group members are likely to pull the individual towards the signal response, but at higher time costs because the response would be slightly delayed. Each line represents the average parameter value of one of the eight evolving populations. (TIFF) [file pcbi.1010442.s008.tiff]

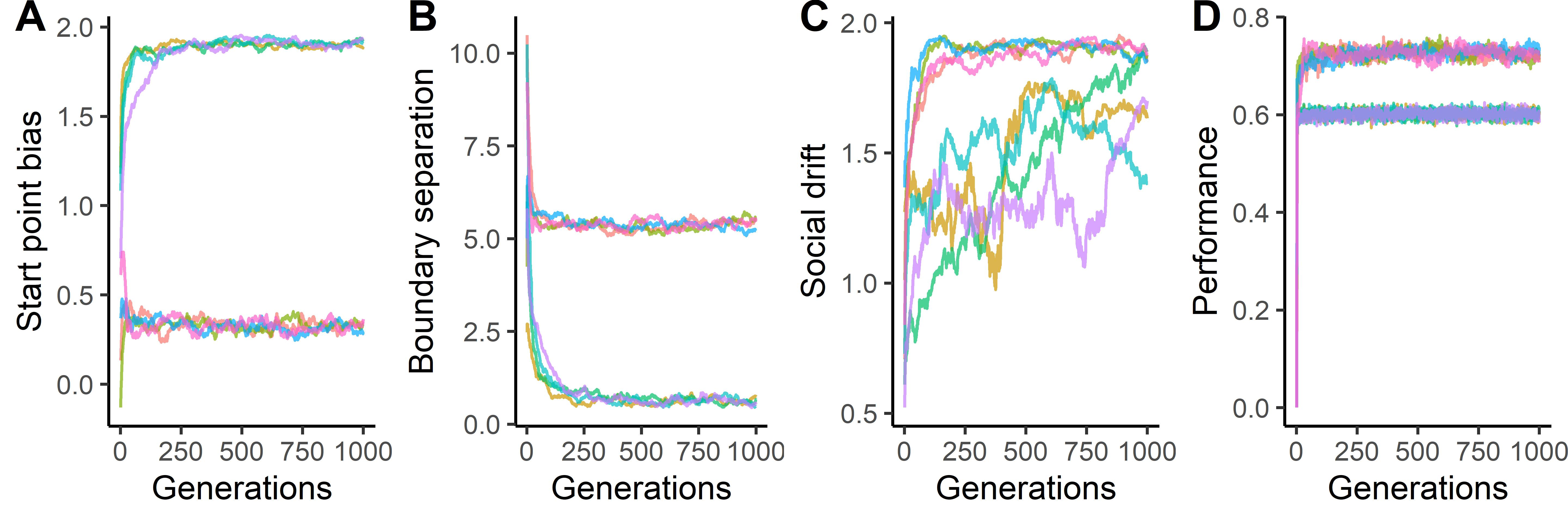

Supplement: S9 Fig — If signal was present in 80% of the simulations, populations with large competitive groups (group size = 50) either converged to be highly biased and make rapid choices or to be less biased with a larger boundary separation. While populations with lower start point bias would, on average, perform better, reducing the start point bias (or increasing the boundary separation) in a highly biased population is likely to be disadvantageous. Less biased individuals would likely be pulled towards the signal response. These results mirror the behaviour found when providing individuals with low quality information (see also S2 and S8 Figs). (TIFF) [file pcbi.1010442.s009.tiff]

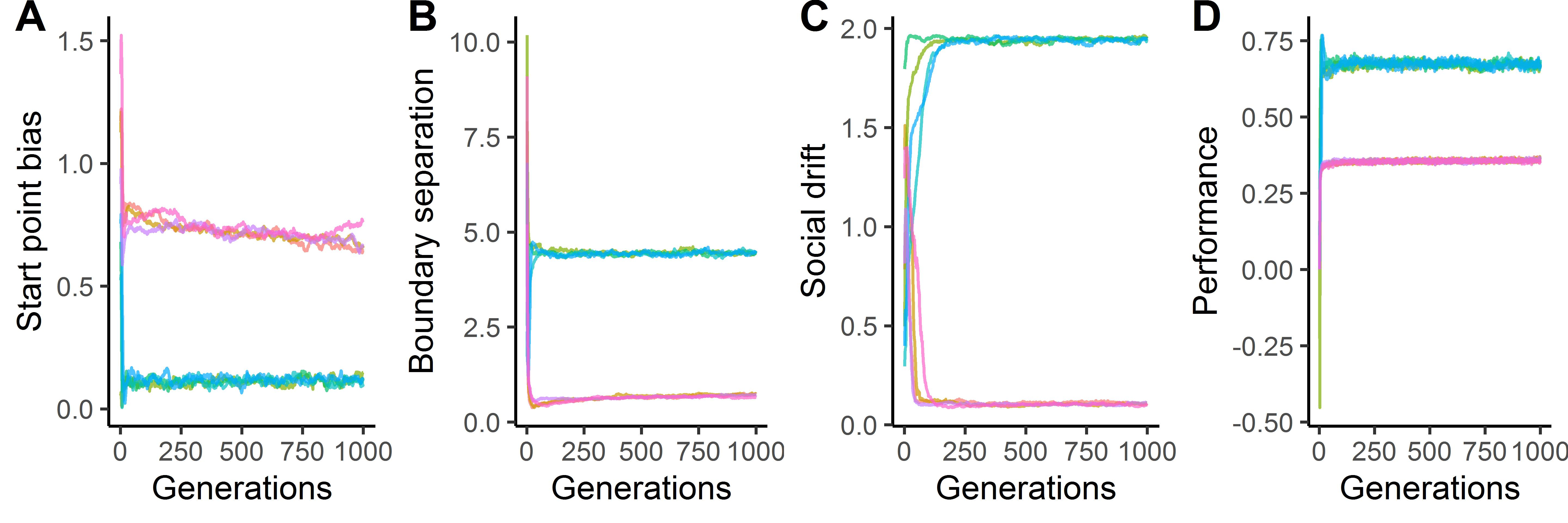

Supplement: S10 Fig — One equilibrium (blue, turquoise, green and light green lines) followed the pattern of our main result, namely (A) low start point bias, (B) medium boundary separation, (C) maximum social drift, and (D) high performance. In the other equilibrium (purple, pink, orange and brown lines), populations converged to a nonsocial behavior with individuals (A) being highly biased, (B) barely waiting, and (C) not incorporating social information, resulting in (D) low performance. This solution is stable, since a less biased individual with larger boundaries is likely to suffer from higher time cost by waiting for and ultimately following other individuals (who almost always choose ‘signal’). Each line represents the average parameter value of one of the eight evolving populations. (TIFF) [file pcbi.1010442.s010.tiff]
